# The Use of Explainable Machine Learning for the Prediction of the Quality of Bulk-Tank Milk in Sheep and Goat Farms

**DOI:** 10.3390/foods13244015

**Published:** 2024-12-12

**Authors:** Daphne T. Lianou, Yiannis Kiouvrekis, Charalambia K. Michael, Natalia G. C. Vasileiou, Ioannis Psomadakis, Antonis P. Politis, Angeliki I. Katsafadou, Eleni I. Katsarou, Maria V. Bourganou, Dimitra V. Liagka, Dimitrios C. Chatzopoulos, Nikolaos M. Solomakos, George C. Fthenakis

**Affiliations:** 1 Veterinary Faculty, University of Thessaly, 43100 Karditsa, Greece; dlianou@vet.uth.gr (D.T.L.); nsolom@uth.gr (N.M.S.); 2 Faculty of Public and One Health, University of Thessaly, 43100 Karditsa, Greecepsomadak@uth.gr (I.P.); agkatsaf@uth.gr (A.I.K.);; 3 School of Veterinary Medicine, European University of Cyprus, Engomi, Nicosia 2404, Cyprus; 4 Faculty of Animal Science, University of Thessaly, 41110 Larissa, Greece

**Keywords:** artificial intelligence, goat, health management, k-nearest neighbour, mastitis, milk composition, milk fat, milk protein, neural network, prediction, random forests, sheep, somatic cell counts, total bacterial counts

## Abstract

The specific objective of the present study was to develop computational models, by means of which predictions could be performed regarding the quality of the bulk-tank milk in dairy sheep and goat farms. Our hypothesis was that use of specific variables related to the health management applied in the farm can facilitate the development of predictions regarding values related to milk quality, specifically for fat content, protein content, fat and protein content combined, somatic cell counts, and total bacterial counts. Bulk-tank milk from 325 sheep and 119 goat farms was collected and evaluated by established techniques for analysis of fat and protein content, for somatic cell counting, and for total bacterial counting. Subsequently, computational models were constructed for the prediction of five target values: (a) fat content, (b) protein content, (c) fat and protein, (d) somatic cell counts, and (e) total bacterial counts, through the use of 21 independent variables related to factors prevalent in the farm. Five machine learning tools were employed: decision trees (18 different models evaluated), random forests (16 models), XGBoost (240 models), k-nearest neighbours (72 models), and neural networks (576 models) (in total, 9220 evaluations were performed). Tools found with the lowest mean absolute percentage error (MAPE) between the five tools used to test predictions for each target value were selected. In sheep farms, for the prediction of protein content, k-nearest neighbours was selected (MAPE: 3.95%); for the prediction of fat and protein content combined, neural networks was selected (6.00%); and for the prediction of somatic cell counts, random forests and k-nearest neighbours were selected (6.55%); no tool provided useful predictions for fat content and for total bacterial counts. In goat farms, for the prediction of protein content, k-nearest neighbours was selected (MAPE: 6.17%); for the prediction of somatic cell counts, random forests and k-nearest neighbours were selected (4.93% and 5.00%); and for the prediction of total bacterial counts, neural networks was selected (8.33%); no tool provided useful prediction models for fat content and for fat and protein content combined. The results of the study will be of interest to farmers, as well as to professionals; the findings will also be useful to dairy processing factories. That way, it will be possible to obtain a distance-aware, rapid, quantitative estimation of the milk output from sheep and goat farms with sufficient data attributes. It will thus become easier to monitor and improve milk quality at the farm level as part of the dairy production chain. Moreover, the findings can support the setup of relevant and appropriate measures and interventions in dairy sheep and goat farms.

## 1. Introduction

Evaluation of the quality of milk produced in sheep or goat farms is important because it reflects upon the quality of dairy products. Good quality raw milk will result in the manufacturing of high-level dairy products and also contribute to maximising milk prices for farmers. Thus, sheep and goat milk should be monitored at every step of the dairy production chain in order to ensure that consumers are offered dairy products that are safe and have a nutritive value.

The quality of the bulk-tank milk produced in sheep and goat farms can be assessed through a variety of laboratory methods. Chemical techniques are employed for measurement of milk constituents, e.g., fat and protein content [1]. Cytological techniques are used to evaluate the somatic cell counts in milk [2]. Finally, for total bacterial counts, microbiological techniques are undertaken [3].

A primary factor that can affect fat and protein content in the milk of sheep and goats is animal nutrition [4,5,6]. Further factors, e.g., animal breed, stage of lactation, milk yield, and mammary infections, can also play a role in the composition of the milk of small ruminants [4,7,8]. The most important determinant of somatic cell counts in the milk of sheep and goats is clinical or subclinical mastitis [9,10,11]. However, factors not related to mammary infection can also influence somatic cell counts, but to a substantially lesser degree; these parameters include animal breed, stage of lactation, milk yield, and type of milking mode (i.e., machine- or hand-milking) [9,10,12]. Total bacterial counts are associated with somatic cell counts (i.e., mammary infections), but may also reflect environmental conditions, e.g., suboptimal cleaning of the milking system [13,14].

The use of machine learning tools can facilitate the estimation of parameters related to the quality of milk of sheep and goats through the provision of a rapid assessment. In the recent past, machine learning approaches have been used for the prediction of the adulteration of milk of sheep or goats through the use of the support vector machines or the neural network tools [15,16,17,18]. The above indicates the scarcity of relevant studies on the application of machine learning methodologies in the assessment of the quality of raw milk of sheep and goats.

In a recently published study, we evaluated machine learning methodologies for the prediction of the presence of mastitis in sheep farms [19]. In that work, we were able to classify farms into those with high or low prevalence of subclinical mastitis based on the evaluation of management- or climate-related variables that prevailed on a farm. Subclinical mastitis leads in and is characterised by alterations in the composition of the milk [20,21], by high somatic cell counts in the milk [10,22], and by increased bacterial isolation from the milk [22,23].

Hence, there is a potential for applying machine learning methodologies for the prediction of the composition of bulk-tank milk and in following up our previous work on the prediction of subclinical mastitis. The specific objective of the present study was to develop computational models, by means of which predictions could be performed regarding the quality of the bulk-tank milk in dairy sheep and goat farms. Our hypothesis was that the use of specific variables related to the conditions prevailing at a farm can facilitate the development of predictions regarding values related to milk quality, specifically for fat content, protein content, fat and protein content combined, somatic cell counts, and total bacterial counts.

## 2. Materials and Methods

### 2.1. Collection of Field and Laboratory Data

**Figure 1:**
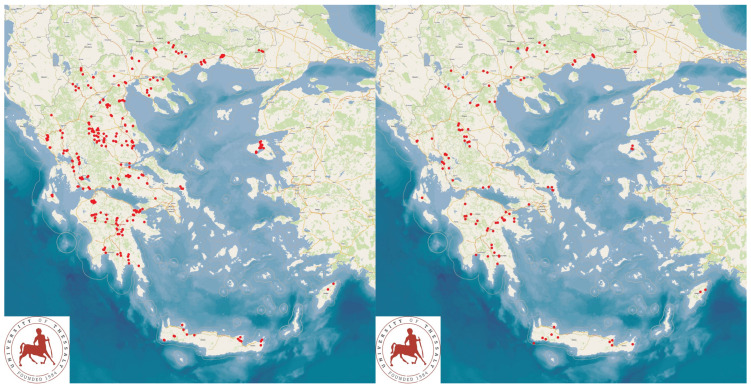
Locations of sheep (**left** map) and goat (**right** map) farms throughout Greece, which were visited for bulk-tank milk sampling.

The data employed for the construction of the computational model were obtained during an extensive countrywide cross-sectional field study performed throughout Greece, specifically in sheep and goat dairy farms. The data were obtained during on-site visits of the researchers to the farms, which were located in all the administrative regions (*n* = 13) of Greece. In total, 325 dairy sheep and 119 dairy goat farms were visited during the field stage of this study (Figure 1). In brief, the farms were selected by collaborating veterinarians active in veterinary practice involving a large amount of clinical work in small ruminants throughout Greece. The farms were selected based on the willingness of the shepherds and goatherds to receive a visit by a university team, participating in an interview by means of a detailed questionnaire and accepting collection of samples from the farm [24].

All the details of the study (including the methodology for selection of the farms) were described by Lianou et al. [25,26]. The farms in the study included in total 110,228 sheep and 30,192 goats. During the on-farm visits, the bulk-milk samples were collected aseptically, directly from the milk cooling tank of each farm. The milk within the tank was consistently mixed (by means of an agitator attached to the lid of the tank) until the cover of the tank was lifted. The samples were collected by using sterile, single-use plastic pipettes, through which the milk samples were withdrawn from the cooling tank. In total, four 20 mL milk samples were collected from the cooling tank of each farm.

Thereafter, an interview was performed with the farmer, by means of a structured questionnaire, through which a detailed mapping of the farm was carried out [24]. The questionnaire included a total of 442 questions. The duration of the interview was on average 64 min. If farmers asked for explanations of the questions, these were provided by the interviewer (author D.T.L.) immediately [24].

Samples were collected from the bulk-tank milk of each farm for subsequent (a) analysis of fat and protein composition, (b) somatic cell counting, and (c) total bacterial counting, as has been described in full detail before [25,26]. Two of the four milk samples collected from each cooling tank were used for evaluation of somatic cell counts and milk content; the other two milk samples were used for total bacterial counting examinations. The first two examinations started within 4 h and the third within 24 h after sample collection. Two sub-samples were created from each of the four samples, and therefore each of the above examinations was carried out four times (in different sub-samples on each occasion). Somatic cell counting and milk composition measurement were performed by using appropriate automatic analysers (Lactoscan SCC and Lactoscan Farm Eco, respectively; Milkotronic Ltd., Nova Zagora, Bulgaria) [25,26]. Total bacterial counts were performed as described before [3].

### 2.2. Dataset Used for the Construction of the Computational Models

The variables used for the construction of the ten computational models (i.e., milk of two different animal species, each with five target values) are described in Table 1. The target values were obtained from the results of the laboratory examinations performed on the bulk-tank milk. The independent variables derived from the information obtained during the on-site farm visits.

### 2.3. Implementation of Machine Learning Algorithms

The scikit-learn library (version 1.4.10 for Python [27]), an open-source library for machine learning that provides various tools for data mining and data analysis tasks, was used to implement machine learning algorithms throughout this work.

### 2.4. Evaluations for Construction of Computational Model by Means of Supervised Learning

Supervised learning can be applied to data that include information, which, after ‘training’, can be used in predictions in new, unseen data. In such cases, the objective is to ‘train’ the model to use patterns in the initial data (‘training dataset’) for making predictions regarding the missing (and requested) information. This simulates a ‘teacher’ providing answers to ‘train’ the computational model.

There are two main types of supervised learning: classification and regression. In classification, predictions refer to the category or group into which the missing information belongs; in regression, predictions refer to values. In the present study, the initial step was to set the type of supervised learning to be applied; in this respect, it was decided to apply regression learning, which would aim to predict the precise values of the target parameters in bulk-tank milk samples.

Subsequently, the development of the computational model involved the selection of the machine learning tool and specific model for achieving optimal predictions. Five different machine learning tools were used in this study to assess their suitability in the prediction in this dataset; these tools were the following: decision trees, random forests, XGBoost, k-nearest neighbours, and neural networks. For each of these five tools, various relevant hyperparameter combinations were tried to assess their suitability. For each combination of hyperparameters, 50 evaluations were conducted among the given dataset, using a mix of resampling, shuffling, and the k-fold cross-validation method (with k = 5).

The data were preprocessed by shuffling and standardising the numerical features to ensure uniform scaling and improve the performance of the model. Specifically, for categorical variables, One-Hot Encoding was applied, which transformed categorical data into a binary matrix, thus allowing the model to interpret and use these features effectively during the ‘training’ stage. That way, it enhanced the compatibility of categorical data with machine learning algorithms.

In each of the 50 evaluations, each dataset was randomly divided into a ‘training’ set and a ‘validation’ set, which always differed between them. Therefore, for the assessment of each tool for each target value, 50 pairs of ‘training’ and ‘validation’ datasets were created and evaluated. On each of these assessments, the model was ‘trained’ on the ‘training’ set, and thereafter its performance was evaluated on the ‘validation’ set. On each different evaluation, the training dataset included 80% of the available data, and the validation dataset included 20% of the available data.

Subsequently, and in order to identify the optimum combination of hyperparameters, the distribution of scores and errors was analysed for each set of hyperparameters on each of the five tools and for each of the ten target values.

**Figure 2:**
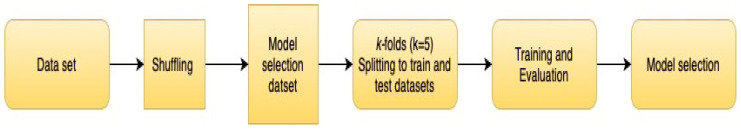
A flow diagram that summarises the procedure of development of machine learning models for the quality of bulk-tank milk in sheep and goat farms.

The procedure for the development of machine learning models is shown in a flow diagram in Figure 2.

### 2.5. Combinations of Hyperparameters Employed in Each Machine Learning Tool

For decision trees, the minimum number of split samples required to be at a leaf node was defined to be equal to 1. The hyperparameter used was the maximum depth of the tree, where the nodes of the model were expanded until all leaves were pure or until all leaves contained fewer than the minimum number of samples required to split an internal node, in this case 2; hence the hyperparameter was set to 3 to 20.

For random forests, a combination of two hyperparameters was employed. More specifically, the number of trees in the forest (10, 100, 200, or 500) and the criteria for measuring split quality (‘squared_error’, ‘absolute_error’, ‘friedman_mse’, or ‘poisson’) were used. With regard to these criteria, the ‘squared_error’ corresponded to the mean squared error (equivalent to variance reduction and minimising L2 loss by averaging values in each terminal node), the ‘absolute_error’ represented the mean absolute error (minimising L1 loss by using the median of each terminal node), the ‘friedman_mse‘ was similar to the ‘squared_error’ with incorporation of Friedman’s improvement score for potential splits, and the ‘poisson’ determined splits based on reductions in Poisson deviance.

For XGBoost, the combinations of the four hyperparameters were as follows. For the L2 regularisation term (*lambda*) on weights, four options were inserted: 1, 2, 5, or 10; for the number of estimators, five options were considered: 800, 900, 1100, 1200, or 1250; for the learning rate, there were three possibilities: 0.08, 0.15, or 0.2; finally, for the maximum depth of the trees, four options were set: 7, 8, 9, or 10.

For k-nearest neighbours, the three hyperparameters used were as follows: *p*, which was assigned values of 1, 2, or 3 (defining the formula of the distance between points in the target values); the number of neighbours (k), which took one of 12 values (1, 2, 3, 4, 5, 6, 7, 8, 9, 10, 15, or 20); and the metric, which was considered to be ‘uniform’ or ‘distance’.

For neural networks, the four hyperparameters used were as follows. The activation function included ‘identity’, ‘logistic’, ‘tanh’ or ‘relu’, the number of hidden layers could take values 2, 5, 10, 20, 50, 100, 200, or 500, the learning rate could take values 0.001, 0.01, 0.1, 0.2, 0.3, or 1 and the solver for weight optimization was ‘lbfgs’, ‘sgd’ or ‘adam’.

In all, 922 evaluations were performed for each of the ten target values under study, i.e., in total 9220 evaluations during this stage of the assessment. Details are in Table 2.

### 2.6. Data Management and Analysis

All data were systematically recorded and organised using Microsoft Excel. The target value ‘fat and protein content’ was produced by adding the two separate values of the fat content and the protein content of each sample. For the analyses, somatic cell counts were transformed to somatic cell scores (SCS); these were calculated as previously described by Wiggans and Shook [28] and Franzoi et al. [29] by using the following formula: SCS = log_2_(SCC/100) + 3. Moreover, total bacterial counts were transformed to log_10_. For these two outcomes, the transformed data were used in the analyses.

The mean absolute percentage error (MAPE) was calculated for each validation dataset for each model tried with each machine learning tool for each target value. Hence, a list of 50 MAPEs was created for each model used in each machine learning tool. The best model produced by each tool was selected for comparison across the five tools employed for the prediction of each target value. Subsequently, for each target value, a comparison was performed among the five machine learning tools by using the MAPEs as the measure of quality. First, the normality of the distribution of MAPEs was assessed by employing the Shapiro–Wilk test. Subsequently, one-way analysis of variance or the Kruskal–Wallis test was used as appropriate in order to compare the MAPEs produced by the best model within each of the five tools; this was followed by Tukey’s HSD or Dunn’s test between the two lower MAPEs. If significant differences were found in the MAPEs between the five tools, the tool with the lowest MAPE was selected for the respective target value; a second tool was additionally selected if no significant difference was indicated between the two tools with the lowest MAPE. Finally, only prediction models with MAPE < 10.0% were deemed useful and were studied further.

Statistical significance was defined at *p* < 0.05.

### 2.7. Analysis of the Importance of the Independent Variables in Predicting the Target Values in the Bulk-Tank Milk—Interpretation of Findings

SHAP (SHapley Additive exPlanations) values analysis was subsequently performed. This analysis could explain the various outputs of computational models based on machine learning methodology, and it was employed with the aim to understand how the individual independent variables used in the various computational models influenced the predictions when using each of the models developed [30]. SHAP quantified feature importance based on principles of game theory and revealed how each feature contributed to the final output of the model. Through the use of the SHAP Python library, SHAP values were calculated for each predicted target value and selected tool(s) [30].

The values found represented the impact of each feature on the prediction’s deviation from the baseline. SHAP determined this impact by assessing how the prediction changed as the features were progressively added to the model in all possible combinations [30].

## 3. Results

### 3.1. Selection of Best Computational Model

The results of the evaluation of the various models (i.e., combinations of hyperparameters) for the selection of the best one (i.e., with the lowest mean absolute percentage error (MAPE)) among each of the five machine learning tools for each of the five target values are in Table S1 (Figure S1) and Table S2 (Figure S2) (in Supplementary Materials), for sheep and goat farms, respectively. For the prediction of the fat content in bulk-tank milk from goat farms, no significant differences were seen among the five tools (*p* = 0.09); for all other target values, the differences among the machine learning tools employed were significant (*p* < 0.025 for all comparisons).

For protein content, fat and protein content, and total bacterial counts in sheep and goat bulk-tank milk, only one tool emerged with significant difference over the others; these tools provided the lowest MAPE for prediction of the respective target values. For fat content in sheep bulk-tank milk and for somatic cell counts in sheep and goat bulk-tank milk, there was no significant difference between the two tools with the lowest MAPE; hence both were selected.

**Figure 3:**
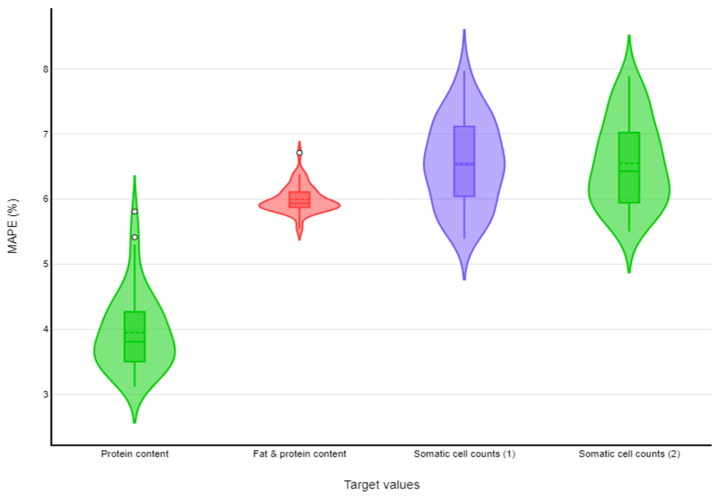
Violin plots for MAPEs < 10.0%, for machine learning tools used for prediction of three target values in bulk-tank milk in dairy sheep farms (green fill: k-nearest neighbours, red fill: neural networks, purple fill: random forests).

**Figure 4:**
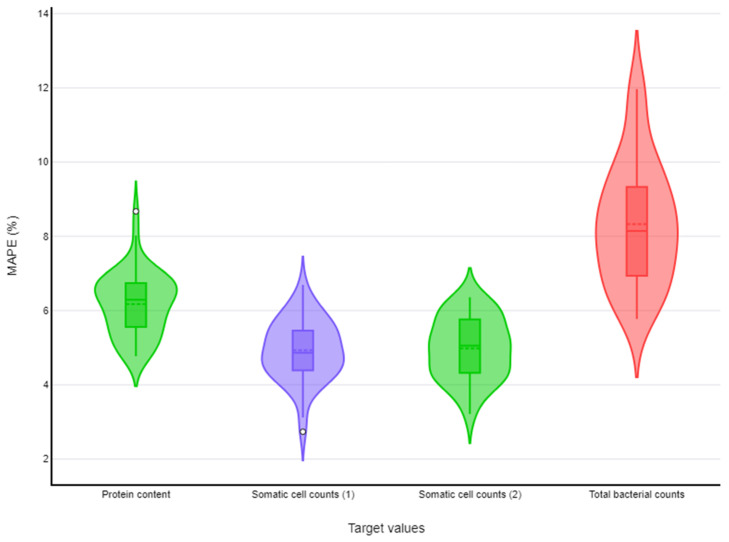
Violin plots for MAPEs < 10.0%, for machine learning tools used for prediction of three target values in bulk-tank milk in dairy goat farms (green fill: k-nearest neighbours, purple fill: random forests, red fill: neural networks).

MAPEs < 10.0% (with upper 95% confidence limit also < 10.0%) were found for three target values in sheep milk: protein content, fat and protein content, and somatic cell counts. MAPEs < 10.0% (with upper 95% confidence limit also < 10.0%) were found also for three target values in goat milk: protein content, somatic cell counts, and total bacterial counts. For both sheep and goat milk, two tools were selected for somatic cell counts, and one tool was selected for each of the other two target values. Details are in Figure 3 and Figure S3 (in Supplementary Materials) and Table 3 (sheep farms) and in Figure 3 and Figure S4 (in Supplementary Materials) and Table 4 (goat farms). Figure 3 and Figure 4 show the distribution of values of MAPEs for target values (*n* = 3 in each case) that achieved MAPEs < 10.0%, in accord with the machine learning tool used for each target value, for sheep and goat farms, respectively.

### 3.2. Results of Analysis of SHAP Values

**Figure 5:**
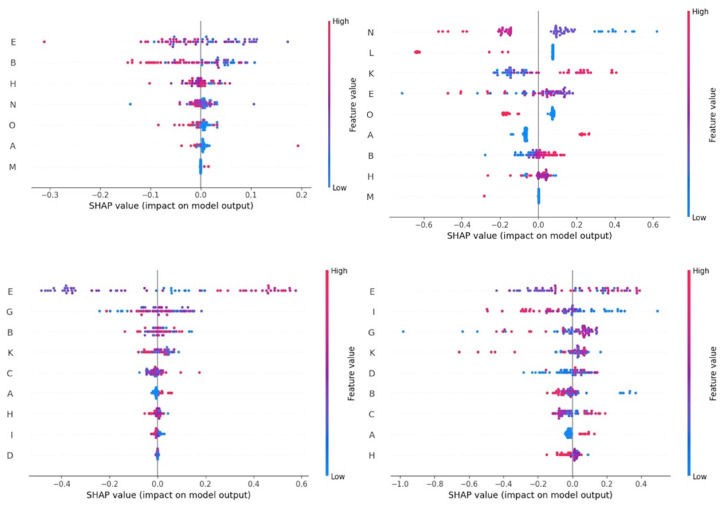
SHapley Additive exPlanations values for the importance of the various independent variables employed in the prediction of three target values (with MAPEs < 10.0%) in bulk-tank milk of sheep farms through computational machine learning models.(clockwise from top left: protein content (k-nearest neighbours), fat and protein content (neural networks), somatic cell counts (random forests), somatic cell counts (k-nearest neighbours); independent variables shown on vertical axis of each graph: A is the presence of milking parlour, B is the month into lactation period at sampling, C is the month of start of milking period, D is the annual incidence rate of clinical mastitis, E is the age of newborns taken away from their dam, G is the age of farmer, H is the education of farmer, I is the body condition score of female animals, K is the animal breed, L is the grazing of animals, P is the provision of concentrates to animals, N is the management system applied in farm, and O is the administration of anthelmintics at last stage of pregnancy; red dots: high feature values, blue dots: low feature values).

**Figure 6:**
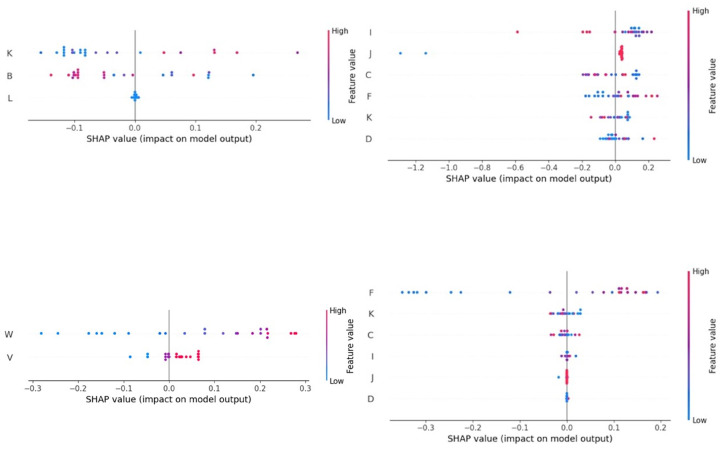
SHapley Additive exPlanations values for the importance of the various independent variables employed in the prediction of three target values (with MAPEs < 10.0%) in bulk-tank milk of goat farms through computational machine learning models. (clockwise from top left: protein content (k-nearest neighbours), somatic cell counts (random forests), somatic cell counts (k-nearest neighbours), total bacterial counts (neural networks); independent variables shown on vertical axis of each graph: B is the month into lactation period at sampling, C is the month of start of milking period, D is the annual incidence rate of clinical mastitis, F is the annual milk production per animal, I is the body condition score of female animals, J is the number of daily milking sessions, K is the animal breed, V is the type of milking parlour, and W is the No. of animals in farm; red dots: high feature values; blue dots: low feature values).

The results of the analysis for SHAP values for the impact of the various independent variables in the prediction of each of the studied outcomes are summarised in Table 5 (sheep farms; details in Figure 5 and Table S3 (in Supplementary Materials)) and Table 6 (goat farms; details in Figure 6 and Table S4 (in Supplementary Materials)). Figure 5 and Figure 6 show the order of SHAP values, which describe the importance of the impact of each independent variable on the prediction’s deviation from the baseline for target values (*n* = 3 in each case) that achieved MAPEs < 10.0%, in accord with the machine learning tool used for each target value, for sheep and goat farms, respectively.

## 4. Discussion

### 4.1. Preamble

The work referred to a study for determining possibilities for the prediction of parameters related to the quality of bulk-tank milk in small ruminant farms. Five parameters, which reflect the chemical, cytological, and microbiological quality of milk, were assessed. Ultimately, predictions for protein content and for somatic cell counts could be proposed for milk in both sheep and goat farms, as well as for the combined fat and protein content in sheep farms and for total bacterial counts in goat farms.

The current study follows on from a previous study on the development of a computational model for the prediction of subclinical mastitis [19]. Both the prevalence of subclinical mastitis and the quality of bulk-tank milk refer to farm-level metrics. Some of the factors that were used for the prediction of the level of subclinical mastitis are also important determinants of parameters related to the quality of bulk-tank milk produced in sheep and goat farms (e.g., month into lactation period at sampling, management system applied in farm, no. of animals in farm, animal breed) [9,10]. Further, previous studies have shown the correspondence between somatic cell counts in bulk-tank milk and the prevalence of subclinical mastitis in sheep or goat farms [11,31,32].

However, our previous work referred to the prediction of the level of subclinical mastitis and focused on a classification problem (i.e., high/low level of prevalence of subclinical mastitis). In contrast, the present study has focused on continuous target values, which required the use of a different methodological approach. In this work, five machine learning tools were used: decision trees, random forests, XGBoost, k-nearest neighbours, and neural networks. This approach aimed to develop a predictive framework to assist with evaluations related to indicators of milk quality. The attempt to set up predictions for five target values intended to provide a complete estimation of the quality of the milk of sheep and goats, with special reference to its hygiene status and, ultimately, to cheesemaking potential.

Bulk-tank milk with high somatic cell counts and total bacterial counts has been linked to reduced cheese yields and to defects in cheese quality (for example, due to increased proteolytic enzyme activity therein [2]). Previous studies on the use of machine learning methodologies to evaluate the quality of the bulk-tank milk in sheep or goat farms involved only the prediction of the adulteration of the raw milk. The originality of the present study refers to the development of computational models for the prediction of five different parameters relevant to the quality of raw milk produced in small ruminant farms.

‘Cheese yield’ is defined as the quantity of cheese produced from a specific quantity of milk and is of particular importance for manufacturers of dairy products, because it determines the financial terms of operation of the business. Several factors that can affect cheese yield have been associated with milk composition [33] and technological traits [34]. In general, the dairy industry estimates the cheesemaking potential of sheep and goat milk on the basis of the content of fat and protein [35], whilst hygiene-related variables (somatic cell counts, total bacterial counts) can also affect it.

The results of the study will be of interest to farmers, as well as to professionals (veterinarians, animal scientists, etc.); the findings will also be useful to dairy processing factories. That way, it will be possible to obtain a distance-aware, rapid, quantitative estimation of the milk output from sheep and goat farms with sufficient data attributes. It will thus become easier to monitor and improve milk quality at the farm level as part of the dairy production chain.

### 4.2. Development of the Models Used

The model was developed based on field data from an extensive study into the quality of milk in sheep and goat farms located across Greece. That way, a variety of management practices applied in small ruminant farms throughout the country were included. In total, combinations of 21 independent variables were employed to predict the five target values related to the quality of bulk-tank milk produced on the farms.

#### 4.2.1. Independent Variables Used in the Development of the Models

These parameters included conditions related to the nutrition of animals (e.g., grazing of animals, provision of concentrates), which is a significant determinant for the fat and the protein contents of the milk produced by sheep and goats [4,36]. The body condition score was also taken into account, as it reflects the nutrition of the animals [37,38] and it has also been associated with the quality of the milk produced by sheep [39]. Other management-related variables (e.g., the animal breed) were included, as these can also influence compositional parameters and somatic cell counts of the milk produced [40,41,42,43]. The inclusion of the stage of lactation among these variables is also noted, given that the composition and the somatic cell counts of milk produced by ewes and does change as the lactation period advances [4,44].

Target values related to milk quality have a multifactorial dependency [10,45,46,47]. This has been considered and is reflected in the independent variables used for the development of the various computational models. The inclusion of a higher number of parameters might have overemphasised patterns and characteristics related to the training data employed in this study [48,49]. A higher number of data in the computational models would also make more difficult the collection of data for considering predictions regarding milk quality.

#### 4.2.2. Selection of Machine Learning Tools

In a published study of the use of machine learning for the prediction of parameters related to milk quality of individual cows, a variety of machine learning approaches was used (partial least square regression, ridge regression, elastic net, model averaging approach, neural network, least absolute shrinkage and selection operator). The precise tool used depended on the target value assessed on each occasion [50].

With regard to the tools employed for the assessment in the current study, random forests, k-nearest neighbours, and neural networks were the tools that were selected for the prediction of the target values. Random forest is an ensemble approach that combines multiple decision trees in order to improve the accuracy and reliability of developed models; generally, this tool outperforms a single tree, can manage large datasets effectively, and can also model non-linear relationships; however, it can be computationally demanding, and the large number of trees can reduce interpretability. k-nearest neighbours is a simple, intuitive algorithm, useful for classification and regression predictions; it is versatile and effective on smaller datasets, as it calculates the distance to every data point, whilst it is also sensitive to feature scaling and can be affected by irrelevant features. Finally, neural networks are flexible models capable of capturing complex, non-linear relationships, which makes them ideal for high-dimensional data; nevertheless, they require significant computational resources in order to achieve optimal performance. The five machine learning tools employed in the present study, namely decision trees, random forests, XGBoost, k-nearest neighbours, and neural networks, have been widely recognised to be sufficient for many machine learning projects due to their diverse capabilities and possibilities for application across a variety of problem types. A summary of the advantages of each of the tools employed and the spectrum of the possibilities of learning approaches are listed below [51].

- Decision trees provide interpretable models and are foundational for ensemble tools like random forests and XGBoost.
- Random forests add robustness in the data analysis by reducing overfitting through ensembling, making the tool versatile for both classification and regression supervised learning.
- XGBoost is a state-of-the-art gradient boosting algorithm that excels in accuracy and efficiency, particularly in structured data.
- k-nearest neighbours refer to a non-parametric and simple tool that leverages local information for effective predictions in spatially related data.
- Neural networks are tools capable of modelling highly complex and non-linear relationships, which extend problem-solving capability to multi-dimensional datasets.

The use of this variety of tools ensured that interpretable and complex tasks, as well as linear and non-linear relationships in the data, were addressed. More specifically, decision trees and random forests offer interpretability and support the understanding of model decisions and feature importance, whilst XGBoost and neural networks handle complex relationships and large-scale datasets. Moreover, the inclusion of random forests and XGBoost among the tools employed ensured a baseline for performance, as these are robust to overfitting and may act as a benchmark to compare against more complex models (e.g., neural networks) [51].

#### 4.2.3. Cross-Validation

The use of a combination of cross-validation with a train–test split of 50 evaluations of datasets is generally considered to be a sound and robust method for model selection [52]. Cross-validation ensures that the model is tested across multiple splits of the data, enhancing its ability to generalise to previously unseen scenarios. By performing and incorporating the results of 50 different train–test splits, this approach captures the inherent variability within the data and ensures that the model’s performance is reliable and not overly dependent on any specific subset. Further, the use of 50 evaluations ensures that the entire dataset contributes to both the training and testing of the model at some point, which increases data efficiency and avoids leaving unused valuable data [53].

#### 4.2.4. Selection of Combinations of Hyperparameters

The final combination of hyperparameters that produced the optimal results for each tool was determined using a greedy search approach [54]. This systematically explores a predefined set of hyperparameter combinations in order to identify the most effective configuration for model performance. For the selection process, all results were thoroughly evaluated based on their performance metrics. Thereafter, the hyperparameter combination with concurrently the lowest mean error and the smallest range of errors across all validation folds was selected. This approach ensured both high accuracy and stability, reducing the likelihood of overfitting or poor generalisation on unseen data. Moreover, care was taken to ensure computational efficiency during the greed search, and the results were cross-validated to confirm the robustness of the selected configurations. This rigorous process helped to maximise the performance and reliability of each machine learning model [53,55].

#### 4.2.5. Selection of Mean Absolute Percentage Error as the Performance Metric

The mean absolute percentage error (MAPE) was selected as the performance metric, because of its intuitive interpretation and practical utility in contexts where relative errors matter more than absolute ones [56]. MAPE expresses the error as a percentage of the actual values, which makes it particularly suitable for the comparison of performance across datasets or situations with varying scales. In cases like the present study (i.e., where predictions involve varying magnitudes), MAPE provides an easily understandable measure of accuracy that is independent of units. Given the study’s emphasis on explainability and practical application, MAPE has also provided a balance between interpretability and relevance to the task [57].

#### 4.2.6. Predictions of Target Values

Our methodologies could not provide a useful prediction for fat content. Possibly, this may reflect the increased sensitivity of fat content mostly to nutritional modifications [58] rather than other management practices. In previous studies, Rajini and Sravani [59] and Samad et al. [60] reported prediction of the level of fat content in cow milk by using classification (i.e., high/low content) rather than regression methodologies.

In contrast, protein content, for which low MAPEs were produced by using the tools employed, is more difficult to manipulate through nutritional interventions and has a more narrow range within which it varies [61]. In a study similar to ours, Frizzarin et al. [50] also reported the use of regression tools for the prediction of the various protein fractions in cow milk. It is also notable that for the prediction of protein content, the k-nearest neighbours tool was found to provide the lowest MAPEs in the milk in both sheep and goat farms.

We also recorded similar issues for the prediction of somatic cell counts. In preliminary evaluations, we used the untransformed somatic cell counts (which varied widely) and obtained MAPEs over 20%. After carrying out the transformation to somatic cell scores (through which the respective target value has a more narrow range of values), the MAPEs decreased to below 7%, which thus could provide a more useful prediction.

#### 4.2.7. Possible Limitations

Possible limitations of the model development include model overfitting and lack of transparency in model decisions. The former was minimised by implementing cross-validation to assess model performance on unseen data and by conducting 50 evaluations among the given dataset using resampling, shuffling, and the k-fold cross-validation method. For the latter, explainable techniques, in this instance SHAP, were also employed; these provided the degree of influence of each of the individual independent variables used in the various models developed of each target value. That way, it has become possible to break down the complex algorithmic decisions into comprehensible components [62].

### 4.3. Importance of the Independent Variables in the Predictions

The identification of the variables with the greatest impact among those requiring simple and easy-to-obtain information (e.g., age of newborns taken away from dam, month into lactation period at sampling, animal breed, month of start of milking period) for the prediction of parameters related to milk quality makes the procedure relatively straightforward for clinicians. The identification of the body condition score among these parameters confirms the value of this parameter as an overall index with relevance to the total production output in small ruminant farms [38].

Key properties of SHAP (SHapley Additive exPlanations) values, making them useful for model interpretation, include (a) accuracy (i.e., provision of an accurate interpretation of the model’s prediction for a specific case), (b) additivity (i.e., provision of efficient computation, even in high-dimensional datasets), (c) consistency (i.e., ensuring similar interpretation of the model’s behaviour, even as the model’s architecture and parameters evolve), and (d) missingness (i.e., confirming that SHAP values are robust for missing data, without irrelevant features potentially distorting the interpretation) [30].

Moreover, the identification of these variables reflects, to a large extent, the changes and adjustments that should be made in the health management of sheep flocks and goat herds in order to improve the quality of the milk produced at the farm, i.e., at the start of the dairy production chain.

### 4.4. Application of the Findings in Small Ruminant Farms—Feasibility and Cost Effectiveness

Machine learning can take into account routinely available data (e.g., information about practices related to health management), with the aim of providing predictions regarding the production output in dairy sheep and goat farms. Moreover, the identification of variables with a major impact on the prediction of the quality of the milk of sheep and goats provides useful information regarding adjustments that need to be made in the farms in order to improve the quality of the milk produced therein. That way, the findings can support the setup of relevant and appropriate measures and interventions in dairy sheep and goat farms.

The results of the study will be of interest to farmers, as well as to professionals (veterinarians, animal scientists, etc.). The findings will also be useful to dairy processing factories.

The advancements in agricultural engineering and the increased application of informatics in agricultural enterprises (including small ruminant farms), allied to the increase in computing power and connectivity—information exchange [63] will support farmers to run their farms and operations more efficiently and with higher productivity [64]. In this context, the present findings can contribute to the building of relevant, practical, and usable decision-support systems and tools [65]. These will be able to integrate the specific features of individual farms, as well as the characteristics of the farmers [66], as part of the development of a ‘digital agriculture’ approach in animal farms.

Notably, however, in Greece, the cost for the development and the maintenance of a machine learning model for milk quality, as described above, can be in the order of EUR 50,000 to 55,000 over the course of the first five years. This sum can rise to EUR 70,000 to 75,000 if aiming to build a framework for the support of future modelling activities, for example, to predict disease patterns in the animals on the farm [19]. This should be compared to the cost of approximately EUR 30,000, which is necessary for installing a brand-new, modern-type, 24-unit milking parlour, which is the most important equipment in a dairy sheep/goat farm. Thus, it becomes evident that the investment and maintenance expenses for a machine learning model are out of scale for individual farms. Hence, the deployment for a model needs to be undertaken for groups of farms, for example, by veterinary practices that wish to provide high-class health management services to dairy farms or by dairy processing factories to monitor and ensure the quality of raw milk produced in farms within their milk zone.

Future research could explore further areas, for example, fine-tuning model parameters and incorporating additional data sources to improve the output of the various models employed. The findings also show that machine learning can contribute to the advancement of sheep and goat farming by addressing challenges, improving decision-making processes, and enhancing clinical work and professional outputs.

## 5. Conclusions

The findings of this study indicate that machine learning algorithms can be employed for the prediction of the quality of milk. Computational prediction models for the quality of milk produced in sheep and goat farms have been developed using field data. These showed excellent performance for some parameters related to milk production: protein content (sheep and goats), fat and protein content (sheep), somatic cell counts (sheep and goats), total bacterial counts (goats). These models can be used as adjunct tools to support the decisions of clinicians in setting up management plans to improve milk quality in small ruminant dairy farms.

## Figures and Tables

**Table 1:** Variables used for the construction of computational models for prediction of bulk-tank milk quality in sheep and goat farms.

	Target Values in Bulk-Tank Milk in Sheep Farms									
Independent Variables	Fat Content (%)		Protein Content (%)		Fat and Protein Content (%)		Somatic Cell Counts (cells mL^−1^)		Total Bacterial Counts (c.f.u. mL^−1^)	
	Type of Farm									
	Sheep	Goats	Sheep	Goats	Sheep	Goats	Sheep	Goats	Sheep	Goats
Month into lactation period at sampling (numerical)		• ^1^	•	•	•	•	•		•	
Month of start of milking period (numerical)							•	•		
Management system applied in farm (categorical)			•		•					
Presence of milking parlour (categorical)		•	•		•	•	•			
Type of milking parlour (categorical)										•
Availability of ventilators in main barn (categorical)									•	
No. of animals in farm (numerical)										•
Animal breed (categorical)	•			•	•	•	•	•		
Grazing of animals (categorical)	•			•	•	•				
Number of daily milkingsessions (categorical)								•		
Cleaning of parlour with water after milking (categorical)									•	
Provision of concentrates to animals (categorical)	•	•	•		•	•				
Age of newborns taken away from dam (numerical)	•	•	•		•	•	•			
Administration of anthelmintics at last stage of pregnancy (categorical)			•		•					
Annual milk production per animal (numerical)								•		
Annual incidence rate of clinical mastitis (numerical)							•	•		
Average number of newborns per dam (numerical)									•	
Body condition score of female animals (numerical)							•	•		
Age of farmer (numerical)							•			
Length of experience of farmer (numerical)									•	
Education of farmer (categorical)			•		•		•		•	

^1^ • represents the variable used in respective computational model.

**Table 2:** Supervised learning tools used, hyperparameters employed for each tool and numbers of models produced during the assessment for each of the ten regression problems under study.

Supervised Learning Tool	Hyperparameters Employed	No. of Different Models Evaluated
Decision trees	(i) minimum number of split samples, (ii) maximum depth of the tree	18
Random forests	(i) number of trees in the forest, (ii) criteria for measuring split quality	16
XGBoost	(i) L2 regularization term (*lambda*), (ii) number of estimators, (iii) learning rate, (iv) maximum depth of the trees	240
k-nearest neighbours	(i) *p*, (ii) number of neighbours (*k*), (iii) distance metric	72
Neural networks	(i) activation function, (ii) hidden layers, (iii) learning rate, (iv) solver	576

**Table 3:** Selected supervised learning tools employed and details of best model selected for each tool and respective mean absolute percentage error (MAPE) (95% confidence interval) produced during the assessment for prediction of five target values related to quality of bulk-tank milk, under evaluation in sheep farms.

Target Value	Supervised Learning Tool	Details of Model Employed	MAPE
Fat content	Random forests	(i) number of trees in the forest = 100, (ii) criteria for measuring split quality = ‘*absolute_error*’	11.37% (11.13%–11.61%)
	k-nearest neighbours	(i) *p* = 1, (ii) number of neighbours (*k*) = 9, (iii) metric = ‘*distance*’	11.39% (11.15%–11.62%)
Protein content	k-nearest neighbours	(i) *p* = 1, (ii) number of neighbours (*k*) = 20, (iii) metric = ‘*uniform*’	3.95% (3.87%–4.03%)
Fat and protein content	Neural networks	(i) activation function = ‘*logistics*’, (ii) hidden layers = 100, (iii) learning rate = 1, (iv) solver = ‘*adam*’	6.00% (5.96%–6.02%)
Somatic cell counts	Random forests	(i) number of trees in the forest = 100, (ii) criteria for measuring split quality = ‘*absolute_error*’	6.55% (6.5%–6.6%)
	k-nearest neighbours	(i) *p* = 1, (ii) number of neighbours (*k*) = 20, (iii) metric = ‘*uniform*’	6.55% (6.46%–6.64%)
Total bacterial counts	Neural networks	(i) activation function = ‘*logistic*’, (ii) hidden layers = 500, (iii) learning rate = 0.20, (iv) solver = ‘*adam*’	10.25% (10.13%–10.37%)

**Table 4:** Selected supervised learning tools employed and details of best model selected for each tool and respective mean absolute percentage error (MAPE) (95% confidence interval) produced during the assessment for prediction of five target values related to quality of bulk-tank milk, under evaluation in goat farms.

Target Value	Supervised Learning Tool	Details of Model Employed	MAPE
Protein content	k-nearest neighbours	(i) *p* = 1, (ii) number of neighbours (*k*) = 8, (iii) metric = ‘*distance*’	6.17% (6.06%–6.29%)
Fat and protein content	Neural networks	(i) activation function = ‘t*anh*’, (ii) hidden layers = 50, (iii) learning rate = 1, (iv) solver = ‘*adam*’	10.62% (10.26%–10.98%)
Somatic cell counts	Random forests	(i) number of trees in the forest = 200, (ii) criteria for measuring split quality = ‘*absolute_error*’	4.93% (4.82%–5.04%)
	k-nearest neighbours	(i) *p* = 1, (ii) number of neighbours (*k*) = 15, (iii) distance metric = ‘*uniform*’	4.98% (4.87%–5.09%)
Total bacterial counts	Neural networks	(i) activation function = ‘*logistic*’, (ii) hidden layers = 20, (iii) learning rate = 0.001, (iv) solver = ‘*adam*’	8.33% (8.11%–8.55%)

**Table 5:** Results of analysis for SHapley Additive exPlanations for the impact of the three most important independent variables in the prediction of target values with MAPEs < 10.0%, in bulk-tank milk of sheep farms.

Target Value/Supervised Learning Tool			
Protein Content/ k-Nearest Neighbours	Fat and Protein Content/ Neural Networks	Somatic Cell Counts/ Random Forests	Somatic Cell Counts/ k-Nearest Neighbours
Age of newborns taken away from dam	Management system applied in farm	Age of newborns taken away from dam	Age of newborns taken away from dam
Month into lactation period at sampling	Grazing of animals	Body condition score of female animals	Age of farmer
Education of farmer	Animal breed	Age of farmer	Month into lactation period at sampling

**Table 6:** Results of analysis for SHapley Additive exPlanations for the impact of the three most important independent variables in the prediction of target values with MAPEs < 10.0%, in bulk-tank milk of goat farms.

Target Value/Supervised Learning Tool			
Protein Content/ k-Nearest Neighbours	Somatic Cell Counts/ Random Forests	Somatic Cell Counts/ k-Nearest Neighbours	Total Bacterial Counts/ Neural Networks
Animal breed	Body condition score of female animals	Annual milk production per animal	No. of animals in farm
Month into lactation period at sampling	Number of daily milking sessions	Animal breed	Type of milking parlour
Grazing of animals	Month of start of milking period	Month of start of milking period	--

## Data Availability

The original contributions presented in this study are included in the article/Supplementary Materials. Further inquiries can be directed to the corresponding author.

## Supplementary Materials

The following supporting information can be downloaded at https://www.mdpi.com/article/10.3390/foods13244015/s1: Table S1: Mean absolute percentage error (MAPE) (95% confidence interval (CI)) for the best model selected among each of five machine learning tools used for the prediction of each of five target values related to the quality of bulk-tank milk in dairy sheep farms; Figure S1: Box and whisker plots of the Mean absolute percentage errors for the best model selected among each of five machine learning tools used for prediction of each of five target values related to quality of bulk-tank milk in dairy sheep farms; Table S2: Mean absolute percentage error (MAPE) (95% confidence interval (CI)) for best model selected among each of five machine learning tools used for prediction of each of five target values related to quality of bulk-tank milk in dairy goat farms; Figure S2: Box and whisker plots of the Mean absolute percentage errors for the best model selected among each of five machine learning tools used for prediction of each of five target values related to quality of bulk-tank milk in dairy goat farms; Figure S3: Box and whisker plots of lowest mean absolute percentage errors for the machine learning tools considered for use for prediction of each of five target values related to quality of bulk-tank milk in dairy sheep farms; Figure S4: Box and whisker plots of lowest Mean absolute percentage errors for the machine learning tools considered for use for prediction of each of four target values related to quality of bulk-tank milk in dairy goat farms; Table S3: Results of analysis for SHapley Additive exPlanations for the impact of the independent variables in the prediction of target values, in bulk-tank milk in sheep farms; Table S4: Results of analysis for SHapley Additive exPlanations for the impact of the independent variables in the prediction of target values, in bulk-tank milk in goat farms.
