# Supplementary material for: The Use of Explainable Machine Learning for the Prediction of the Quality of Bulk-Tank Milk in Sheep and Goat Farms
Source: Foods. 2024 Dec 12;13(24):4015. doi: 10.3390/foods13244015 (PMC11726918; doi:10.3390/foods13244015)
Supplement: Supplementary file 1 [file foods-13-04015-s001.zip › foods-3315528-supplementary.pdf]

# The Use of Explainable Machine Learning for the Prediction of the Quality of Bulk-Tank Milk in Sheep and Goat Farms

**Daphne T. Lianou, Yiannis Kiouvrekis, Charalambia K. Michael, Natalia G. C. Vasileiou, Ioannis Psomadakis, Antonis P. Politis, Angeliki I. Katsafadou, Eleni I. Katsarou, Maria V. Bourganou, Dimitra V. Liagka, Dimitrios C. Chatzopoulos, Nikolaos M. Solomakos and George C. Fthenakis**

**Table S1.** Mean absolute percentage error (MAPE) (95% confidence interval (CI)) for best model selected among each of five machine learning tools used for prediction of each of five target values related to quality of bulk-tank milk in dairy sheep farms.

| Supervised learning tool | Hyperparameter combination selected | MAPE (95% CI) |
|---|---|---|
| | Fat content | |
| Decision trees | (i) minimum number of split samples = 1, (ii) maximum depth of the tree = 4 | 12.10% (11.9%-12.3%) |
| Random Forests | (i) number of trees in the forest = 100, (ii) criteria for measuring split quality = '*absolute_error*' | 11.37% (11.1%-11.6%) |
| XGBoost | (i) L2 regularization term (lambda) = 2, (ii) number of estimators = 1250, (iii) learning rate = 0.08, (iv) maximum depth of the trees = 8 | 12.27% (12.0%-12.5%) |
| k-Nearest neighbours | (i) p = 1, (ii) number of neighbours (*k*) = 9, (iii) metric = '*distance*' | 11.39% (11.2%-11.6%) |
| Neural networks | (i) activation function = '*logistic*', (ii) hidden layers = 200, (iii) learning rate = 1, (iv) solver = 'a*dam*' | 11.48% (11.3%-11.7%) |
| | *p* value | 0.024 |
| | Protein content | |
| Decision trees | (i) minimum number of split samples = 1, (ii) maximum depth of the tree = 3 | 4.60% (4.5%-4.7%) |
| Random Forests | (i) number of trees in the forest = 10, (ii) criteria for measuring split quality = '*poisson*' | 4.54% (4.4%-4.6%) |
| XGBoost | (i) L2 regularization term (lambda) = 1, (ii) number of estimators = 800, (iii) learning rate = 0.20, (iv) maximum depth of the trees = 7 | 4.60% (4.5%-4.7%) |
| k-Nearest neighbours | (i) p = 1, (ii) number of neighbours (*k*) = 20, (iii) metric = '*uniform*' | 3.95% (3.9%-4.0%) |
| Neural networks | (i) activation function = '*identity*', (ii) hidden layers = 2, (iii) learning rate = 1, (iv) solver = '*lbfgs*' | 4.33% (4.3%-4.4%) |
| | *p* value | < 0.0001 |
| | Fat & protein content | |
| Decision trees | (i) minimum number of split samples = 1, (ii) maximum depth of the tree = 20 | 7.45% (7.3%-7.6%) |
| Random Forests | (i) number of trees in the forest = 500, (ii) criteria for measuring split quality = '*absolute_error*' | 6.74% (6.6%-6.8%) |

| | | |
|---|---|---|
| XGBoost | (i) L2 regularization term (lambda) = 10, (ii) number of estimators = 800, (iii) learning rate = 0.08, (iv) maximum depth of the trees = 9 | 7.54% (7.4%-7.7%) |
| k-Nearest neighbours | (i) p = 1, (ii) number of neighbours (*k*) = 15, (iii) metric = '*uniform*' | 6.35% (6.3%-6.4%) |
| Neural networks | (i) activation function = '*logistics*', (ii) hidden layers = 100, (iii) learning rate = 1, (iv) solver = '*adam*' | 6.00% (5.9%-6.0%) |
| | *p* value | < 0.0001 |
| Somatic cell counts | | |
| Decision trees | (i) minimum number of split samples = 1, (ii) maximum depth of the tree = 20 | 7.87% (7.8%-8.0%) |
| Random Forests | (i) number of trees in the forest = 100, (ii) criteria for measuring split quality = '*absolute_error*' | 6.55% (6.5%-6.6%) |
| XGBoost | (i) L2 regularization term (lambda) = 10, (ii) number of estimators = 800, (iii) learning rate = 0.20, (iv) maximum depth of the trees = 10 | 8.62% (8.5%-8.7%) |
| k-Nearest neighbours | (i) p = 1, (ii) number of neighbours (*k*) = 20, (iii) metric = '*uniform*' | 6.55% (6.5%-6.6%) |
| Neural networks | (i) activation function = = '*logistic*', (ii) hidden layers = 500, (iii) learning rate = 0.20, (iv) solver = '*adam*' | 6.88% (6.8%-7.0%) |
| | *p* value | < 0.0001 |
| Total bacterial counts | | |
| Decision trees | (i) minimum number of split samples = 1, (ii) maximum depth of the tree = 20 | 11.56% (11.4%-11.7%) |
| Random Forests | (i) number of trees in the forest = 100, (ii) criteria for measuring split quality = '*absolute_error*' | 10.82% (10.7%-11.0%) |
| XGBoost | (i) L2 regularization term (lambda) = 10, (ii) number of estimators = 800, (iii) learning rate = 0.20, (iv) maximum depth of the trees = 10 | 12.00% (11.9%-12.2%) |
| k-Nearest neighbours | (i) p = 1, (ii) number of neighbours (*k*) = 20, (iii) distance metric = '*uniform*' | 10.32% (10.2%-10.4%) |
| Neural networks | (i) activation function = '*logistic*', (ii) hidden layers = 500, (iii) learning rate = 0.20, (iv) solver = '*adam*' | 10.25% (10.1%-10.4%) |
| | *p* value | < 0.0001 |

**Figure S1.** Box and whisker plots of the Mean absolute percentage errors for the best model selected among each of five machine learning tools [1] used for prediction of each of five target values [2] related to quality of bulk-tank milk in dairy sheep farms.

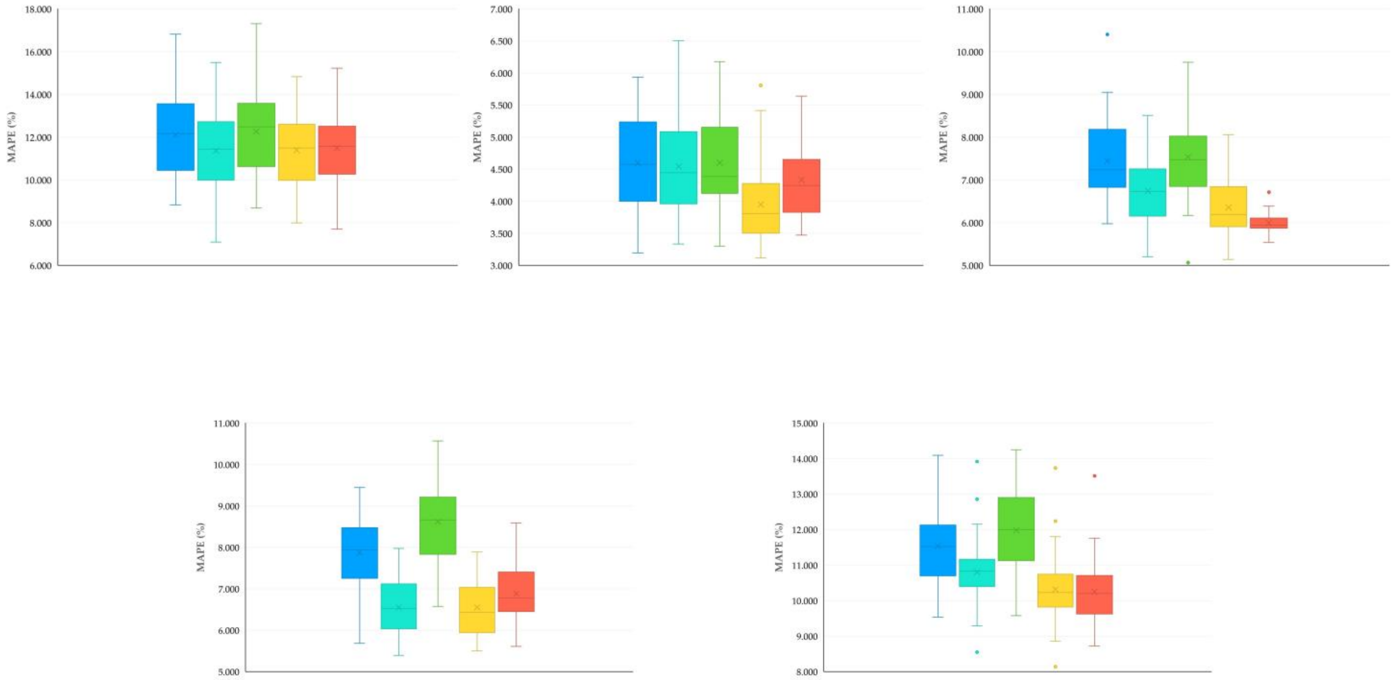


1: blue: Decision trees, teal: Random Forests, green: XGBoost, yellow: k-Nearest neighbours, red: Neural networks.

2: clockwise from top left plot: fat content, protein content, fat & protein content, somatic cell counts, total bacterial counts.

**Table S2.** Mean absolute percentage error (MAPE) (95% confidence interval (CI)) for best model selected among each of five machine learning tools used for prediction of each of five target values related to quality of bulk-tank milk in dairy goat farms.

| **Supervised learning tool** | **Hyperparameter combination selected** | **MAPE (95% CI)** |
|---|---|---|
| | Fat content | |
| Decision trees | (i) minimum number of split samples = 1, (ii) maximum depth of the tree = 4 | 23.64% (22.9%-24.4%) |
| Random Forests | (i) number of trees in the forest = 200, (ii) criteria for measuring split quality = '*absolute_error*' | 22.54% (21.8%-23.2%) |
| XGBoost | (i) L2 regularization term (lambda) = 5, (ii) number of estimators = 800, (iii) learning rate = 0.15, (iv) maximum depth of the trees = 8 | 23.50% (22.8%-24.2%) |
| k-Nearest neighbours | (i) p = 3, (ii) number of neighbours (*k*) = 20, (iii) metric = '*distance*' | 21.50% (20.8%-22.2%) |
| Neural networks | (i) activation function = '*logistic*', (ii) hidden layers = 5, (iii) learning rate = 1, (iv) solver = '*adam*' | 21.52% (21.0%-22.1%) |
| | *p* value | 0.09 |
| | Protein content | |
| Decision trees | (i) minimum number of split samples = 1, (ii) maximum depth of the tree = 3 | 7.02% (6.9%-7.2%) |
| Random Forests | (i) number of trees in the forest = 200, (ii) criteria for measuring split quality = '*absolute_error*' | 6.45% (6.3%-6.6%) |
| XGBoost | (i) L2 regularization term (lambda) = 5, (ii) number of estimators = 800, (iii) learning rate = 0.20, (iv) maximum depth of the trees = 8 | 6.96% (6.8%-7.1%) |
| k-Nearest neighbours | (i) p = 1, (ii) number of neighbours (*k*) = 8, (iii) metric = '*distance*' | 6.17% (6.1%-6.3%) |
| Neural networks | (i) activation function = '*logistic*', (ii) hidden layers = 500, (iii) learning rate = 0.01, (iv) solver = '*lbfgs*' | 6.9% (6.7%-7.1%) |
| | *p* value | 0.0002 |
| | Fat & protein content | |
| Decision trees | (i) minimum number of split samples = 1, (ii) maximum depth of the tree = 20 | 14.93% (14.6%-15.3%) |
| Random Forests | (i) number of trees in the forest = 500, (ii) criteria for measuring split quality = '*absolute_error*' | 13.88% (13.6%-14.2%) |
| XGBoost | (i) L2 regularization term (lambda) = 5, (ii) number of estimators = 800, (iii) learning rate = 0.08, (iv) maximum depth of the trees = 9 | 16.36% (16.0%-16.7%) |
| k-Nearest neighbours | (i) p = 2, (ii) number of neighbours (*k*) = 20, (iii) metric = '*uniform*' | 13.41% (13.2%-13.6%) |
| Neural networks | (i) activation function = '*tanh*', (ii) hidden layers = 50, (iii) learning rate = 1, (iv) solver = '*adam*' | 10.62% (10.3%-11.0%) |
| | *p* value | < 0.0001 |
| | Somatic cell counts | |
| Decision trees | (i) minimum number of split samples = 1, (ii) maximum depth of the tree = 20 | 5.85% (5.7%-6.0%) |

| | | |
|---|---|---|
| Random Forests | (i) number of trees in the forest = 200,<br>(ii) criteria for measuring split quality = '*absolute_error*' | 4.93% (4.8%-5.0%) |
| XGBoost | (i) L2 regularization term (lambda) = 5, (ii) number of estimators = 800,<br>(iii) learning rate = 0.20, (iv) maximum depth of the trees = 10 | 6.89% (6.8%-7.0%) |
| k-Nearest neighbours | (i) p = 1, (ii) number of neighbours (*k*) = 15, (iii) distance metric = '*uniform*' | 4.98% (4.9%-5.1%) |
| Neural networks | (i) activation function = '*logistic*', (ii) hidden layers = 20,<br>(iii) learning rate = 0.001, (iv) solver = '*adam*' | 5.97% (5.9%-6.1%) |
| | *p* value | < 0.0001 |
| | Total bacterial counts | |
| Decision trees | (i) minimum number of split samples = 1, (ii) maximum depth of the tree = 20 | 9.28% (9.1%-9.5%) |
| Random Forests | (i) number of trees in the forest = 200,<br>(ii) criteria for measuring split quality = '*absolute_error*' | 9.50% (9.3%-9.7%) |
| XGBoost | (i) L2 regularization term (lambda) = 5, (ii) number of estimators = 800,<br>(iii) learning rate = 0.20, (iv) maximum depth of the trees = 10 | 10.93% (10.7%-11.2%) |
| k-Nearest neighbours | (i) p = 1, (ii) number of neighbours (*k*) = 15, (iii) distance metric = '*uniform*' | 8.47% (8.2%-8.7%) |
| Neural networks | (i) activation function = '*logistic*', (ii) hidden layers = 20,<br>(iii) learning rate = 0.001, (iv) solver = '*adam*' | 8.33% (8.1%-8.5%) |
| | *p* value | < 0.0001 |

**Figure S2.** Box and whisker plots of the Mean absolute percentage errors for the best model selected among each of five machine learning tools [1] used for prediction of each of five target values [2] related to quality of bulk-tank milk in dairy goat farms.

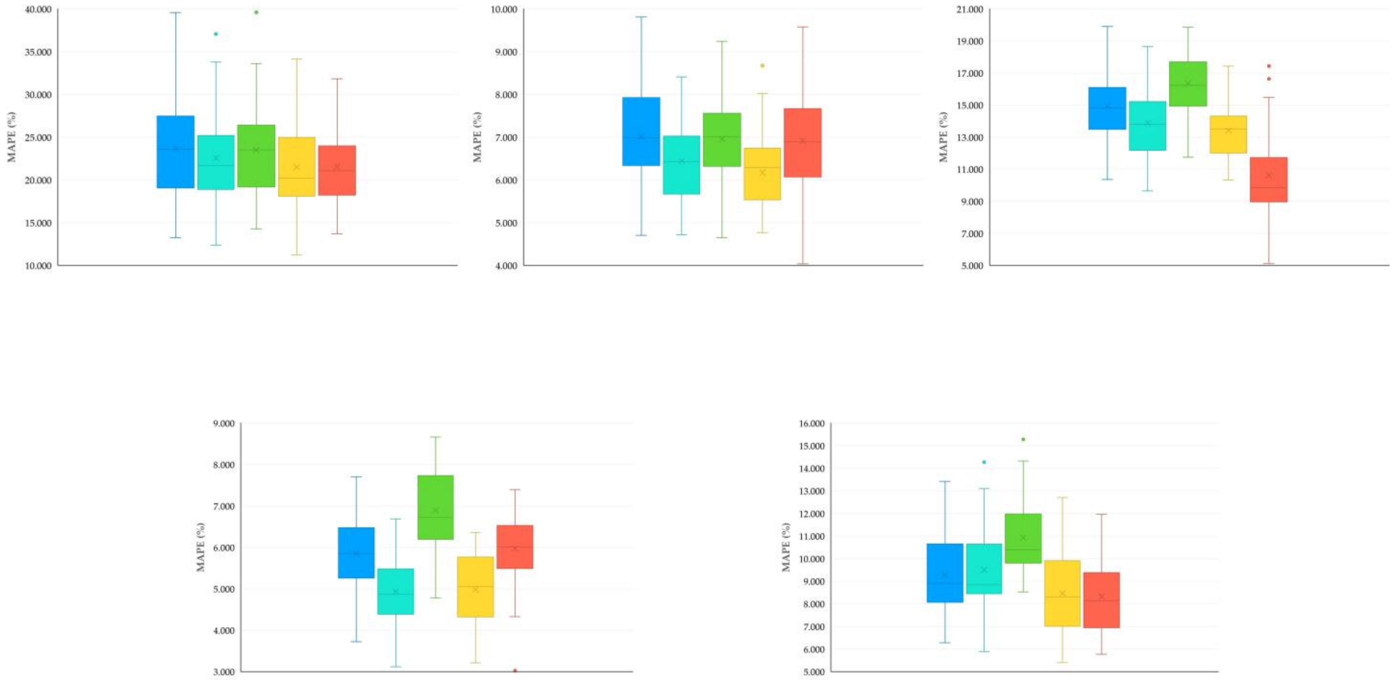


1. blue: Decision trees, teal: Random Forests, green: XGBoost, yellow: k-Nearest neighbours, red: Neural networks.

2. clockwise from top left plot: fat content, protein content, fat & protein content, somatic cell counts, total bacterial counts.

**Figure S3.** Box and whisker plots of lowest Mean absolute percentage errors for the machine learning tools [1] considered for use for prediction of each of five target values [2] related to quality of bulk-tank milk in dairy sheep farms.

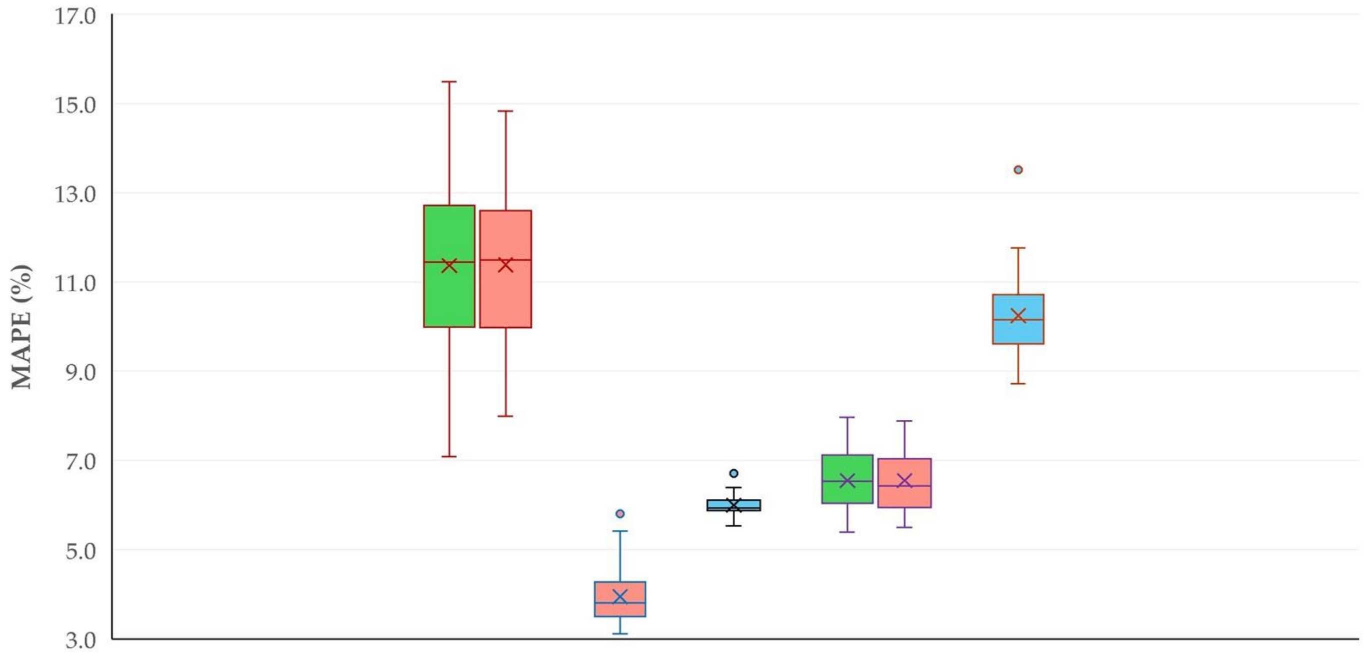


1: green fill: Random forests, red fill: k-Nearest neighbours, blue fill: Neural networks.

2: red outline: fat content, blue outline: protein content, black outline: fat & protein content, purple outline: somatic cell counts, brown outline: total bacterial counts.

**Figure S4.** Box and whisker plots of lowest Mean absolute percentage errors for the machine learning tools [1] considered for use for prediction of each of four target values [2] related to quality of bulk-tank milk in dairy goat farms.

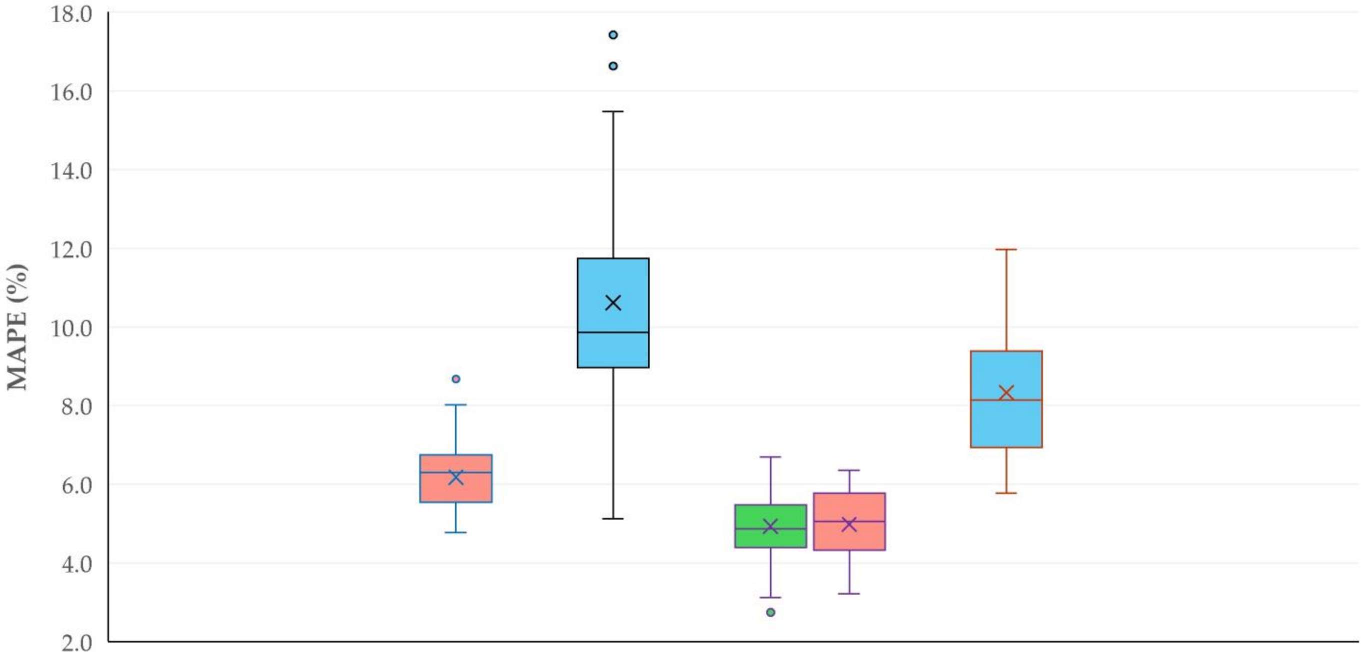


1: green fill: Random forests, red fill: k-Nearest neighbours, blue fill: Neural networks.

2: blue outline: protein content, black outline: fat & protein content, purple outline: somatic cell counts, brown outline: total bacterial counts.

**Table S3.** Results of analysis for SHapley Additive exPlanations for the impact of the independent variables in the prediction of five target values, in bulk-tank milk in sheep farms.

| Target value / Supervised learning tool | | | | | | |
|---|---|---|---|---|---|---|
| Fat content / Random forests | Fat content / k-Nearest neighbours | Protein content / k-Nearest neighbours | Fat & Protein Content / Neural networks | Somatic Cell Counts / Random Forests | Somatic Cell Counts / k-Nearest neighbours | Total bacterial counts / Neural networks |
| Animal breed | Animal breed | Age of newborns taken away from dam | Management system applied in farm | Age of newborns taken away from dam | Age of newborns taken away from dam | Average number of newborns per dam |
| Age of newborns taken away from dam | Age of newborns taken away from dam | Month into lactation period at sampling | Grazing of animals | Body condition score of female animals | Age of farmer | Month into lactation period at sampling |
| Grazing of animals | Grazing of animals | Education of farmer | Animal breed | Age of farmer | Month into lactation period at sampling | Length of experience of farmer |
| Provision of concentrates to animals | Provision of concentrates to animals | Management system applied in farm | Age of newborns taken away from dam | Animal breed | Animal breed | Availability of ventilators in main barn |
| | | Administration of anthelmintics at last stage of pregnancy | Administration of anthelmintics at last stage of pregnancy | Annual incidence rate of clinical mastitis | Month of start of milking period | Education of farmer |
| | | Presence of milking parlour | Presence of milking parlour | Month into lactation period at sampling | Presence of milking parlour | Parlour cleaning with water after milking |
| | | Provision of concentrates to animals | Month into lactation period at sampling | Month of start of milking period | Education of farmer | |
| | | | Education of farmer | Presence of milking parlour | Body condition score of female animals | |
| | | | Provision of concentrates to animals | Education of farmer | Annual incidence rate of clinical mastitis | |

**Table S4.** Results of analysis for SHapley Additive exPlanations for the impact of the independent variables in the prediction of four target values, in bulk-tank milk in goat farms.

| **Target value / Supervised learning tool** | | | | |
|---|---|---|---|---|
| Protein content / k-Nearest neighbours | Fat & Protein Content / Neural networks | Somatic Cell Counts / Random Forests | Somatic Cell Counts / k-Nearest neighbours | Total bacterial counts / Neural networks |
| Animal breed | Month into lactation period at sampling | Body condition score of female animals | Annual milk production per animal | No. of animals in farm |
| Month into lactation period at sampling | Animal breed | Number of daily milking sessions | Animal breed | Type of milking parlour |
| Grazing of animals | Age of newborns taken away from dam | Month of start of milking period | Month of start of milking period | |
| | Grazing of animals | Annual milk production per animal | Body condition score of female animals | |
| | Provision of concentrates to animals | Animal breed | Number of daily milking sessions | |
| | Presence of milking parlour | Annual incidence rate of clinical mastitis | Annual incidence rate of clinical mastitis | |
